# Supplementary material for: Polydopamine Doping and Pyrolysis of Cellulose Nanofiber Paper for Fabrication of Three-Dimensional Nanocarbon with Improved Yield and Capacitive Performances
Source: Nanomaterials (Basel). 2021 Nov 30;11(12):3249. doi: 10.3390/nano11123249 (PMC8707509; doi:10.3390/nano11123249)
Supplement: Supplementary file 1 [file nanomaterials-11-03249-s001.zip › nanomaterials-1469503-supplementary.pdf]

Supplementary Materials

Polydopamine Doping and Pyrolysis of Cellulose Nanofiber Paper for Fabrication of Three-Dimensional Nanocarbon with Improved Yield and Capacitive Performances

Luting Zhu *, Kojiro Uetani, Masaya Nogi and Hirotaka Koga *

SANKEN (The Institute of Scientific and Industrial Research), Osaka University, 8-1 Mihogaoka, Osaka, Ibaraki 567-0047, Japan; uetani@eco.sanken.osaka-u.ac.jp (K.U.); nogi@eco.sanken.osaka-u.ac.jp (M.N.)

* Correspondence: sharollzhu@eco.sanken.osaka-u.ac.jp (L.Z.); hkoga@eco.sanken.osaka-u.ac.jp (H.K.); Tel.: +81-6-6879-8442 (L.Z. & H.K.)

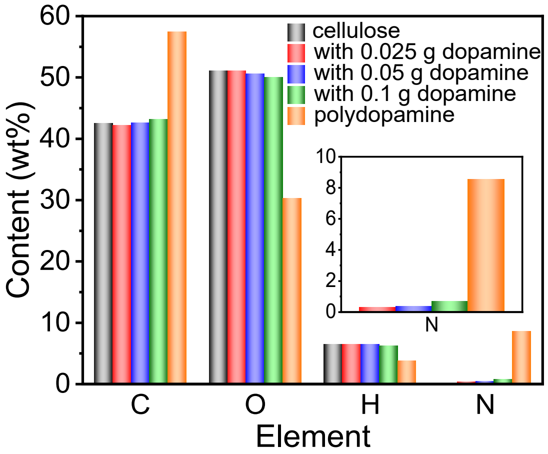

Figure S1. Elemental content of cellulose nanofiber papers with varying dopamine content.

The polydopamine content in the cellulose nanofiber paper can be calculated by elemental analysis. Since the cellulose molecule has no N, its presence in the polydopamine-doped cellulose nanofiber paper can be attributed to the polydopamine component. Based on the calculations, adding 0.025 g, 0.05 g, and 0.1 g dopamine into 0.4 g cellulose resulted in 3.4 wt%, 4.3 wt%, and 8.2 wt% of polydopamine doping, respectively.

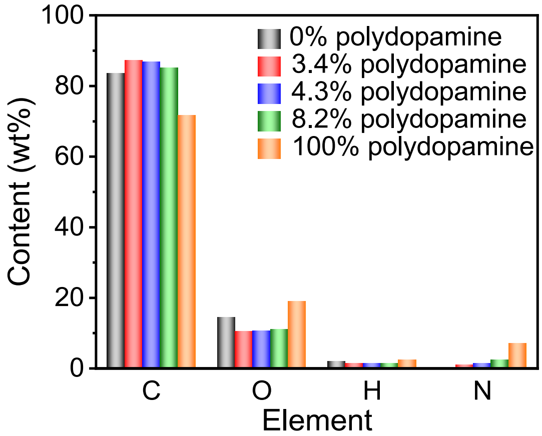

Figure S2. Elemental content of the original and the polydopamine-doped cellulose nanofiber papers pyrolyzed at 700 °C.

The carbon yield of the original and polydopamine-doped cellulose nanofiber paper pyrolyzed at 700 °C was estimated from their carbon content before and after pyrolysis. Notably, polydopamine doping improved the carbon yield (from 16.8% to 28.9%) of the pyrolyzed cellulose nanofiber paper (**Figure 2d**). Moreover, the carbon yields of the pyrolyzed polydopamine-doped cellulose nanofiber paper were higher than the estimated ones; the carbon yields of the pyrolyzed polydopamine-doped cellulose nanofiber paper were 26.1%, 26.4% and 28.9% for the polydopamine contents of 3.4%, 4.3%, and 8.2%, respectively, which were higher than those (19.1%, 19.8%, and 22.7%) estimated from the original cellulose nanofiber paper and neat polydopamine after pyrolysis.

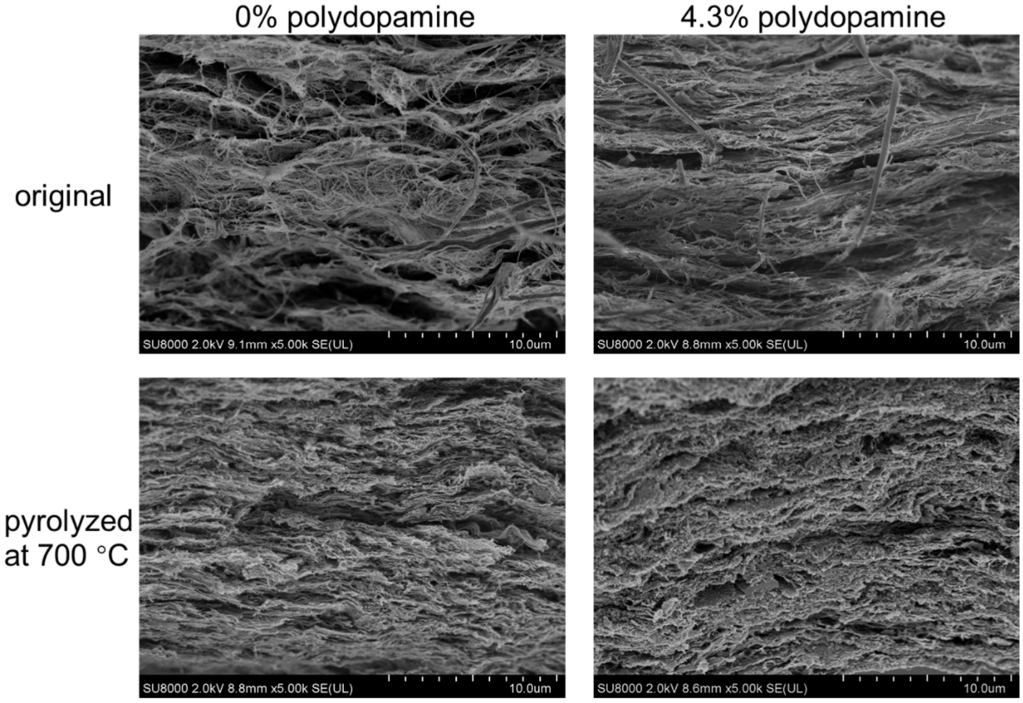

Figure S3. Cross-sectional field emission scanning electron microscopy images of the original and the pyrolyzed cellulose nanofiber paper with different polydopamine content (0% and 4.3%).

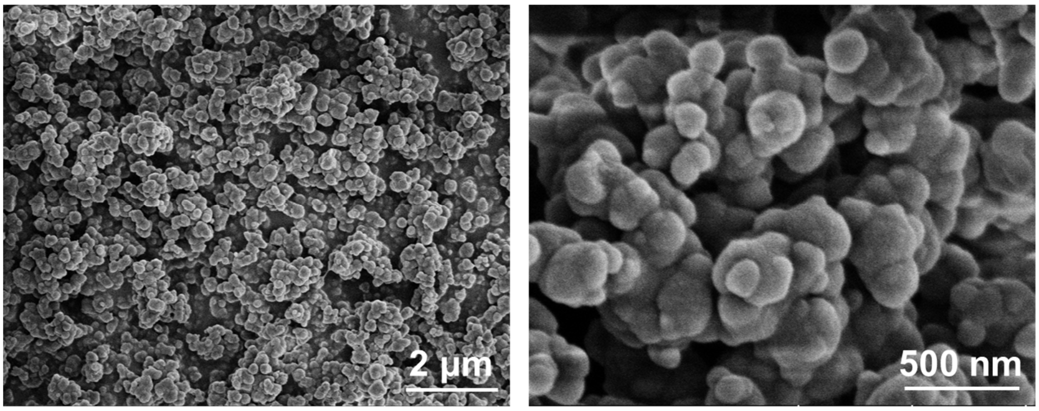

Figure S4. Field emission scanning electron microscopy images of neat polydopamine.

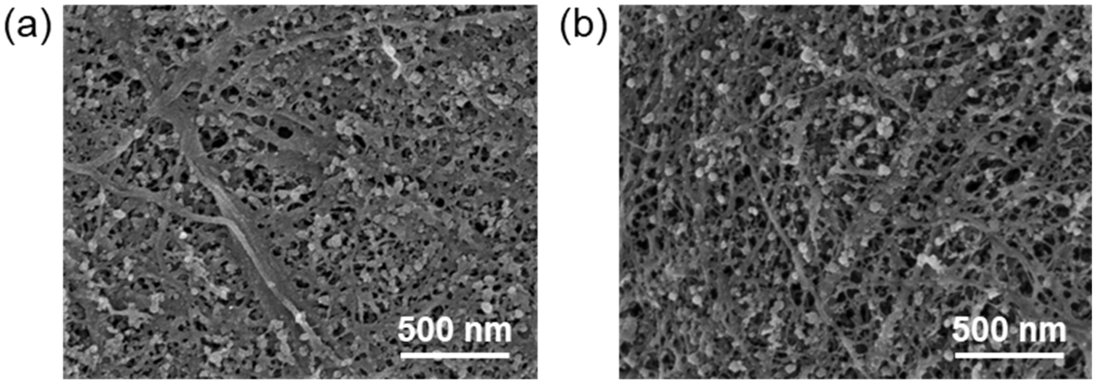

Figure S5. Field emission scanning electron microscopy images of electrode (pyrolyzed 4.3% polydopamine-doped cellulose nanofiber paper) (**a**) before and (**b**) after electrochemical tests.

The porous nanostructures of the pyrolyzed polydopamine-doped cellulose nanofiber paper were observed before and after electrochemical tests through field emission scanning electron microscopy. Prior to the observation, the electrode was washed with distilled water after the electrochemical tests to remove excess KOH from the electrolyte, and it was then oven dried at 60 °C for 2 h.

It has been reported that K^+ (electrolyte ion) with a large radius can cause the structural deformation of the electrode during the insertion and extraction processes [1,2]. In this study, however, the pyrolyzed polydopamine-doped cellulose nanofiber paper maintained its porous nanostructures even after electrochemical tests, suggesting that the pyrolyzed polydopamine nanoparticles were immobilized well on the pyrolyzed cellulose nanofiber networks.

Table S1. Specific capacitance values of cellulose-derived porous carbon materials.

Materials	Surface Area (m^2 g^{-1})	Additive in Electrode	Electrolyte	Capacitance (F g^{-1})	Ref.
Carbonized cellulose areogel	646	acetylene black and binder	1 M H_2SO_4	195 (0.1 A g^{-1})	[3]
Cellulose-derived (3.2%) N-doped carbon	472	no	1 M H_2SO_4	193 (0.5 A g^{-1})	[4]
Wood-derived carbon nanofiber aerogel	689	binder	2 M H_2SO_4	140 (0.5 A g^{-1})	[5]
Cotton fabric derived (9.0%) N-doped carbon	617	carbon black and binder	6 M KOH	180 (0.5 A g^{-1})	[6]
Cellulose nanocrystal/ cellulose nanofibril derived carbon film	1244	carbon black and binder	2 M KOH	170 (0.5 A g^{-1})	[7]
Pyrolyzed polydopamine-doped (1.3% N) cellulose nanofiber paper	617	no	6 M KOH	200 (0.5 A g^{-1})	This work

The pyrolyzed polydopamine-doped cellulose nanofiber paper provided higher specific capacitance than previously reported cellulose-derived nanocarbon materials (**Table S1**). It also presented higher specific capacitance than previously reported biomass-derived carbons such as lignin- (168 F g^{-1} at 10 mV s^{-1}) [8], starch- (144 F g^{-1} at 0.625 A g^{-1}) [9], and alginate- (183 F g^{-1} at 0.5 A g^{-1}) [10] derived porous carbon materials.

References

1. Li, J.; Zhuang, N.; Xie, J.; Zhu, Y.; Lai, H.; Qin, W.; Javed, M.S.; Xie, W.; Mai, W. Carboxymethyl cellulose binder greatly stabilizes porous hollow carbon submicrospheres in capacitive K-Ion storage. *ACS Appl. Mater. Interfaces* **2019**, *11*, 15581–15590.
2. Xu, X.; Mai, B.; Liu, Z.; Ji, S.; Hu, R.; Ouyang, L.; Liu, J.; Zhu, M. Self-sacrificial template-directed ZnSe@C as high performance anode for potassium-ion batteries. Chem. Eng. J. 2020, 387, 124061.
3. Tian, X.; Zhu, S.; Peng, J.; Zuo, Y.; Wang, G.; Guo, X.; Zhao, N.; Ma, Y.; Ma, L. Synthesis of micro- and meso-porous carbon derived from cellulose as an electrode material for supercapacitors. *Electrochim. Acta* **2017**, *241*, 170–178.
4. Chen, Z.; Peng, X.; Zhang, X.; Jing, S.; Zhong, L.; Sun, R. Facile synthesis of cellulose-based carbon with tunable N content for potential supercapacitor application. *Carbohydr. Polym.* **2017**, *170*, 107–116.
5. Li, S.C.; Hu, B.C.; Ding, Y.W.; Liang, H.W.; Li, C.; Yu, Z.Y.; Wu, Z.Y.; Chen, W.S.; Yu, S.H. Wood-derived ultrathin carbon nanofiber aerogels. *Angew. Chemie - Int. Ed.* **2018**, *57*, 7085–7090.
6. Chen, L.; Ji, T.; Mu, L.; Zhu, J. Cotton fabric derived hierarchically porous carbon and nitrogen doping for sustainable capacitor electrode. *Carbon* **2017**, *111*, 839–848.
7. Li, Z.; Ahadi, K.; Jiang, K.; Ahvazi, B.; Li, P.; Anyia, A.O.; Cadien, K.; Thundat, T. Freestanding hierarchical porous carbon film derived from hybrid nanocellulose for high-power supercapacitors. *Nano Res.* **2017**, *10*, 1847–1860.
8. Zhang, W.; Lin, H.; Lin, Z.; Yin, J.; Lu, H.; Liu, D.; Zhao, M. 3 D Hierarchical Porous Carbon for Supercapacitors Prepared from Lignin through a Facile Template-Free Method. *ChemSusChem* **2015**, *8*, 2114–2122.
9. Pang, L.; Zou, B.; Zou, Y.; Han, X.; Cao, L.; Wang, W.; Guo, Y. A new route for the fabrication of corn starch-based porous carbon as electrochemical supercapacitor electrode material. *Colloids Surfaces A Physicochem. Eng. Asp.* **2016**, *504*, 26–33.
10. Chen, W.; Luo, M.; Yang, K.; Zhou, X. Simple pyrolysis of alginate-based hydrogel cross-linked by bivalent ions into highly porous carbons for energy storage. *Int. J. Biol. Macromol.* **2020**, *158*, 265–274.
